# A Study on the Construction of Emotion Recognition Based on Multimodal Information Fusion in English Learning Cooperative and Competitive Mode

**DOI:** 10.3389/fpsyg.2021.767844

**Published:** 2021-11-24

**Authors:** Haihua Tu

**Affiliations:** College of Foreign Languages, Shaoxing University, Shaoxing, China

**Keywords:** multi-mode information fusion, English learning, cooperation and competition, team learning model, emotion recognition

## Abstract

With the development of science and education, English learning has become increasingly important. In the past, English learning was mainly based on missionaries, and students were not very motivated to learn. The purpose of this article is to use the English cooperative model to improve the enthusiasm and initiative of students in learning, and to improve the efficiency of students in learning English. A team learning model based on the game is proposed. This article constructs a cooperative and competitive model of English learning based on multimodal information fusion. The main manifestation is that students form groups in small groups, and there is a competitive relationship between the groups. The competition among students in learning is the common interest of the entire group, so that the overall interests of each student will be more competitive. This article refers to the main body association model in the literature to adjust English grammar, vocabulary, and language perception ability: learn together in team communication to improve students' multifaceted abilities. Finally, a questionnaire was designed. The results show that after changing the English team learning mode and optimizing the English team learning support system of the students' English learning team, the English learning cooperation and competition model based on multimode information fusion proposed in this article can improve the learning effect by 55%-60%. In all English teaching, the two dimensions of professional knowledge and English ability training are not mutually orthogonal and mutually exclusive, but mutually supportive and interdependent. To form an effective teaching model of “student-centered and teacher-led,” active and rich communication and feedback in the classroom are the keys, and they also help to form a gradual teaching and learning cycle.

## Introduction

Cooperative learning in English teaching requires the cooperation of teachers and students, mutual help, and teamwork, while integrating the competitive model into cooperative learning, to stimulate students' enthusiasm for learning and enhance the learning effect. The traditional English learning mode is mainly based on teacher's preaching. Teachers become the main body of the classroom, especially for language subjects, students with poor basic ability, it is difficult to integrate into the classroom, and students often feel boring. The game-based English teaching model has emerged and achieved good results. Although the quality of scientific research output is discussed in this article, it is reasonable to produce different results because of the differences in the selection of research objects and indicators. Inspired by the research, we can continue to explore whether the U-shaped relationship between interdisciplinary and team output quality depends on other factors, such as team member recognition or team communication.

Through literature review and empirical analysis, Eknc and others believe that unplanned management and team member conflict are the main causes of research failure (Eknc, 2020). Siddiqui's research points out that the lack of face-to-face communication will lead to members who are geographically far away from the core of the team to feel confused about team goals and tasks (Siddiqui and Georgiadis, 2020). Freedberg believes that game design in the teaching process can trigger users' learning motivation, generate learning achievements, enrich learning experience, and correct learning attitudes (Freedberg et al., 2019). Liao first put forward the word “Gamification,” but the use of gamification design thinking to improve the experience of Internet products has gradually become popular since 2010 (Liao et al., 2018). Corell proposed that the game design is a “people-oriented design,” which means that in the non-game context, game thinking and interesting elements are used to stimulate user behavior and meet human psychological motivation and needs (Corell et al., 2018).

In terms of educational methods, Qinchen found that proper autonomous learning (such as video and game interaction) can effectively improve students' performance by changing teaching strategies and comparing students' performance changes before and after learning, and can change the way of autonomous learning according to different learning objectives (Cao et al., 2021). Cano found that although there was no significant difference between the game group and the traditional group in learning environment and curriculum attitude, the game group was better than the traditional group in group cohesion, score, and team evaluation (Cano and Villón, 2018). Lobov believes that informal institutional differences will hinder communication and exchange between the two sides of cooperation, which will have a negative impact on transnational cooperation and innovation (Lobov et al., 2020). Li W believes that appropriate informal institutional distance will bring differentiated complementary knowledge and provides impetus for cooperative innovation, and the relationship between informal institutional distance and Transnational Cooperative Innovation is inverted U-shaped (Li and Gu, 2020). Through empirical analysis, Zidan et al. found that the impact of informal institutional distance on Transnational Cooperative Innovation is not significant (Zidan et al., 2019). The above studies all recognize the disadvantages of the traditional non-team learning model, but most of them evaluate the learning situation from the perspective of English learners' behavior and do not form a complete set of English learning models, so it is the lack of research value for the wide applicability of learners.

This article constructs a cooperative and competitive model of English learning based on multimodal information fusion. The main manifestation is that students form groups in small groups, and there is a competitive relationship between the groups, it can stimulate students' interest in learning, give play to their enthusiasm for learning, and enhance the interaction in learning. The competition among students in learning is the common interest of the entire group, so that the overall interests of each student will be more competitive. This article refers to the main body association model in the literature to adjust English grammar, vocabulary, and language perception ability: learn together in team communication to improve students' multifaceted abilities. Finally, a questionnaire was designed.

## English Learning Status and Team Learning Evaluation Model

### Current Situation of Traditional English Classroom

**Figure 1:**
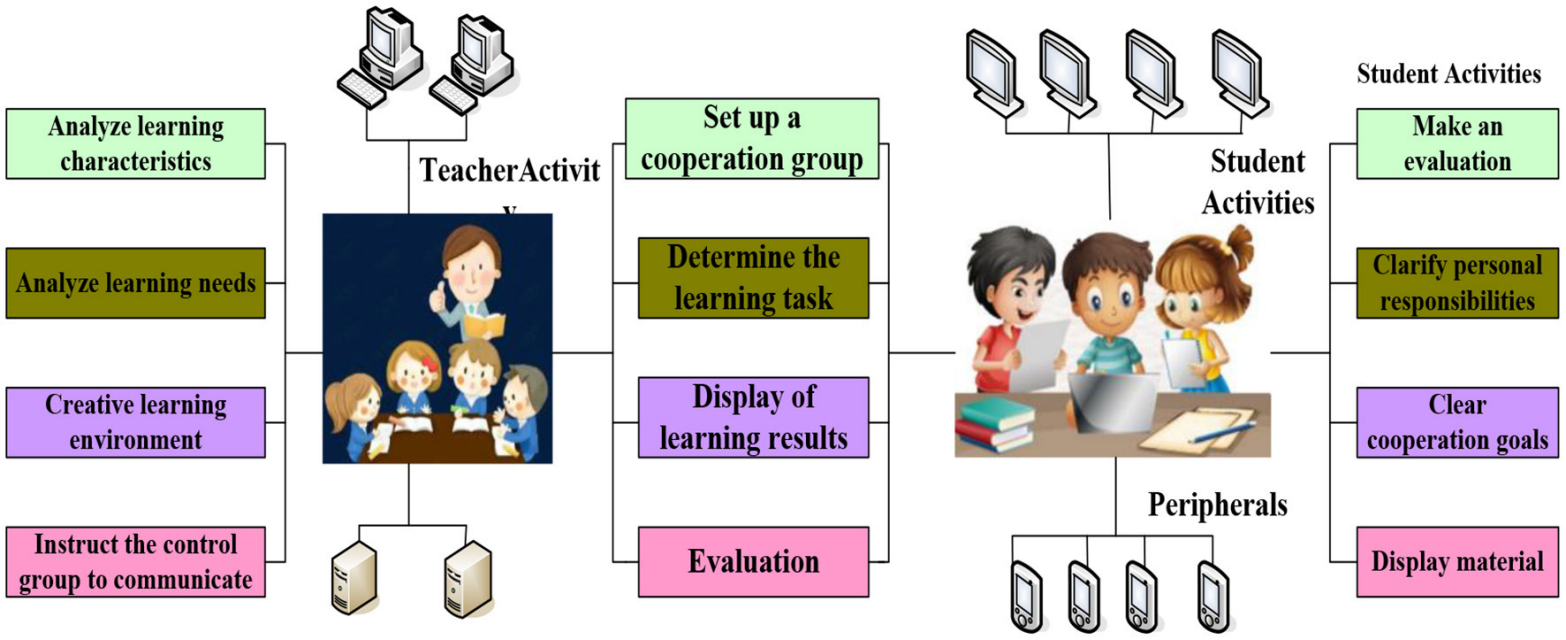
The way of cooperation between teachers and students.

The traditional formative assessment of the classroom is facing many difficulties in the process of implementation. On the one hand, the traditional formative assessment techniques and strategies put forward high requirements for teachers, who need to have evidence thinking, and observe and record students' process performance while teaching. This increases the difficulty of implementing formative assessment (Mohammed et al., 2018). On the other hand, traditional formative assessment techniques such as classroom observation records, activity records, and portfolio use, paper materials, and the process of filling, receiving, and sorting is very time-consuming, and it is difficult to store assessment materials such as learning products (Wariyo, 2019). In recent years, although some classroom teaching systems or learning management systems have created conditions for recording process data in electronic form, they have not fundamentally solved the difficulties in the implementation of formative assessment. Because these evaluation techniques only focus on the data collection link of evaluation, they fail to systematically solve the problem of teachers' implementation of formative evaluation (Lobov et al., 2020). With the development of information technology in the classroom, flipped classroom, problem-solving learning, project-based learning, and other new teaching modes have emerged one after another, which are student-centered and mainly aimed at cultivating students' high-level abilities (such as collaborative inquiry abilities, innovation abilities, etc.) (Hamzah and Nasri, 2020). The learning tasks of these classrooms are oriented to the formal modeling and intelligent calculation of classroom teaching evaluation. With the transformation of classroom teaching from solidifying single teacher instruction to emphasizing group cooperation and participation, how to implement formative assessment in the classroom with cooperative learning as the basic feature has become an urgent problem to be solved in teaching evaluation reform (Liao et al., 2019). With the support of intelligent technology, formal modeling can deconstruct the complex and changeable classroom teaching process to form a mathematical model; intelligent computing can evaluate students' learning state through algorithms and generate teaching auxiliary information according to teaching principles (Telaumbanua et al., 2020). The combination of the two can promote the effective integration of human intelligence and machine intelligence, and form a classroom evaluation mechanism of human-computer cooperation (Ghodbane and Achachi, 2019). The general framework of formal modeling and intelligent computing for classroom teaching evaluation consists of four parts: perception and storage of teaching and learning behavior, construction of teaching and learning behavior evaluation model, intelligent computing of teaching and learning status, and generation of teaching auxiliary information. The first two parts focus on the representation of educational situations and problems, which is the key step of formal modeling; the latter two parts focus on the realization of the specific technical routes, which is the embodiment of the specific process and function of intelligent computing (Emerson et al., 2018). Based on the perception and storage of teaching and learning behavior, the whole system determines the output of the model by constructing the evaluation model; then, it introduces an intelligent algorithm to calculate the model to achieve the evaluation of teaching and learning status; finally, according to the corresponding teaching principles, it automatically generates the information to assist teachers in classroom teaching evaluation (Mustafa, 2018). The further improvement of the general architecture, human-computer collaborative teaching, and evaluation mechanism requires researchers to work with teachers to “design together.” And activities are often complex and difficult, so group cooperation to solve problems and jointly create learning products are common ways (Vellayan et al., 2020). At the same time, the development of such complex teaching activities requires teachers to adjust teaching strategies in time according to the students' learning progress. All of these put forward higher requirements for the intelligence of classroom teaching evaluation. How to implement formative assessment in the classroom characterized by collaborative learning has become an urgent problem to be solved in the reform of classroom teaching evaluation (Nievecela and Ortega, 2019). Some researchers propose to use formal modeling and intelligent computing to help and support teachers more systematically and to form a man-machine collaborative classroom evaluation mechanism (Carrillo et al., 2019). Formal modeling methods, originated from software engineering, aim at modeling representation based on combing the software development process and realizing the standardization of software design, development, and verification. The formal modeling of classroom teaching needs to collect data according to software and hardware equipment, establish a data set according to certain standards, and on this basis, make clear the input and output status of the model according to the actual classroom needs (Nelli and Hartati, 2018). Intelligent computing is the process of introducing machine learning, deep learning, and other intelligent algorithms to calculate the input data after modeling, get the output state represented by mathematics, and use this to analyze learners' potential learning characteristics and rules (Juliati, 2019). In classroom teaching evaluation, formal modeling is to make a symbolic representations of classroom teaching and learning behavior to form a mathematical model; intelligent computing is to analyze and calculate the represented teaching and learning behavior to assist teachers in classroom teaching evaluation (Ghufron and Ermawati, 2018). The linkage of the two can not only realize automation in the process of evidence collection and explanation of classroom teaching evaluation, but also give teachers support in the process of implementing teaching behavior, so it has become an important direction of classroom teaching evaluation reform. The way of cooperation between teachers and students is shown in Figure 1.

### Typical Classroom Teaching Evaluation System and Architecture

**Figure 2:**
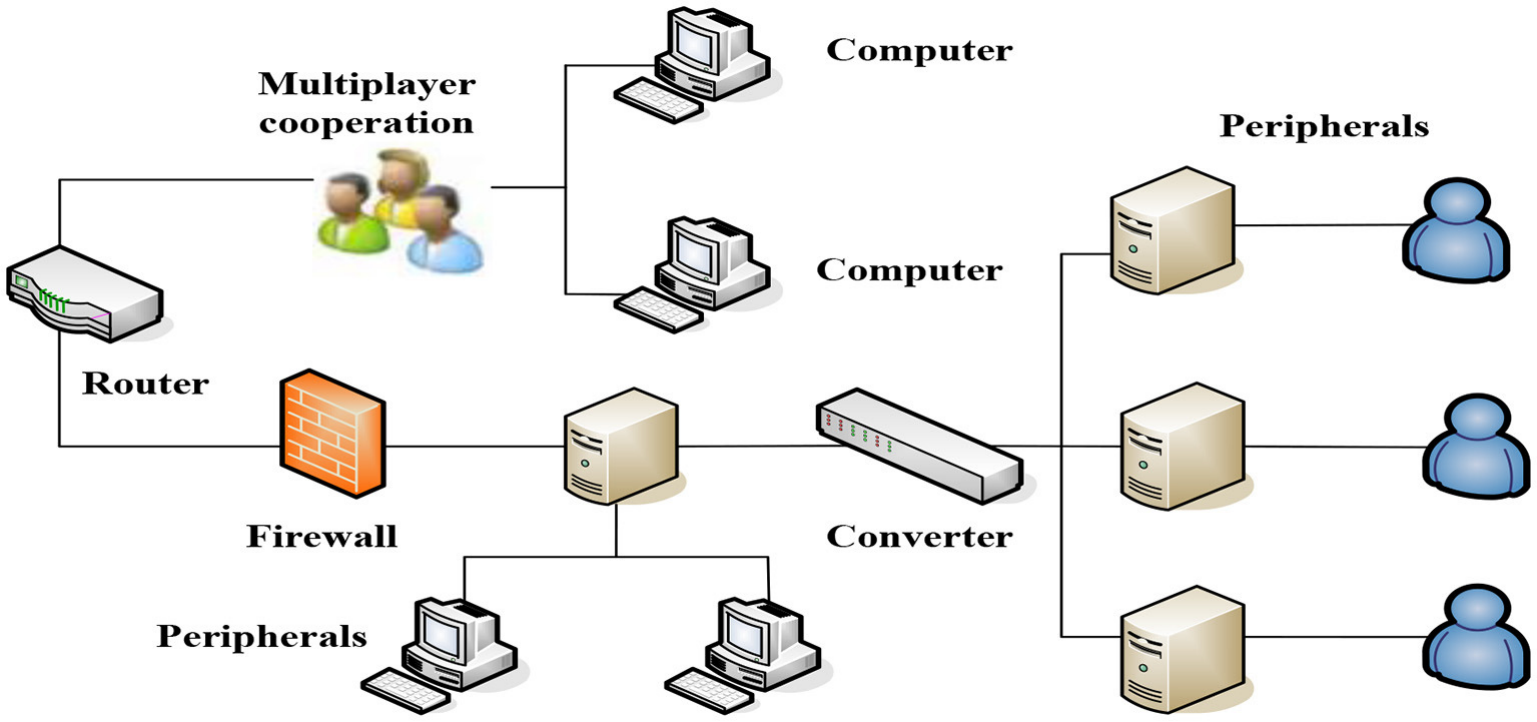
The teacher's teaching framework.

With the support of intelligent technology, the classroom management system develops rapidly. Considering that the classroom management system to provide classroom teaching evaluation must have the basic functions of real-time analysis, evaluation of learning process, and providing feedback information for teachers, this article summarizes the fact intelligent teaching management system through research. The fact system takes students' operation behavior and answer records as input data and provides information and guidance for teachers in cognition, learning status, and cooperation with others through certain calculation methods. On the computing path, these systems follow the ideas of formal modeling and intelligent computing, and not only focus on students' individual learning behaviors and group cooperative learning behavior in the classroom, but also provide support for the transformation of individual, group, and class activities. Input data are as follows: student's operation behavior, answer record student's operation behavior, answer record student's real-time code, group learning product output state, student's personal cognitive state and abnormal state, student's personal cognitive state, problem-solving state, and cooperation state, students' individual cognitive state, students' individual problem-solving progress and learning engagement state, group problem-solving progress, cooperation state, group cognitive state, problem-solving progress, and cooperation state. In the actual classroom, students may switch between individual learning and cooperative learning, so the intelligent system for classroom teaching evaluation needs to pay attention to the two learning processes at the same time. The teacher's teaching framework is shown in Figure 2.

### Deduction and Evaluation Model of English Competitive Learning

**Figure 3:**
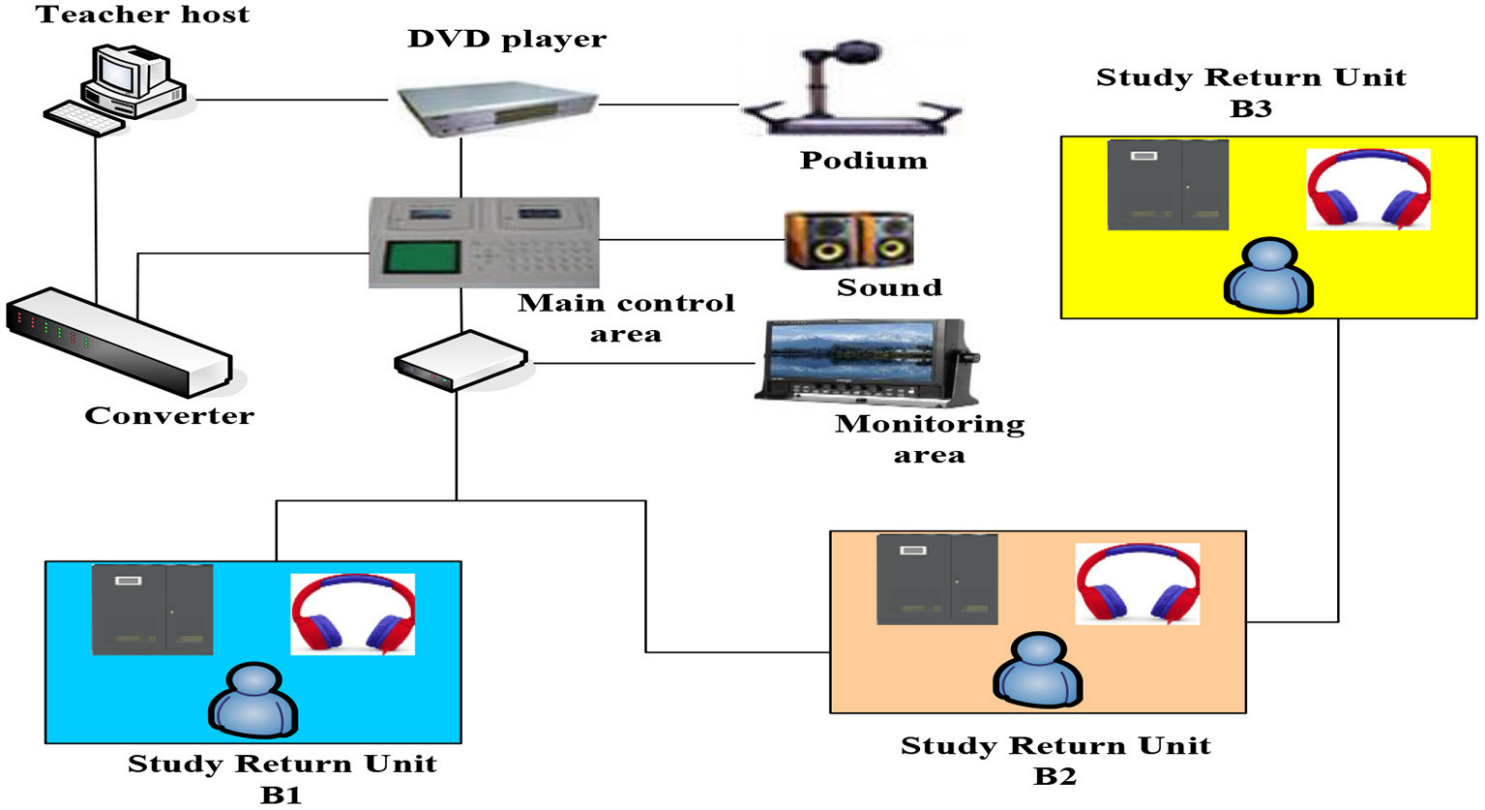
The English competitive learning model.

This article proposes an overall prediction model for cooperative prediction in English learning. First, through the cooperative network of English learning participants, the embedded vector representation of English learning participants is obtained by using the network representation learning method. The English competitive learning model is shown in Figure 3.


(1) $$\begin{array}{l} A_{\lim}=f\left(x\right)=\underset{j\subset Q}{\sum }c_{j}\frac{x_{j}}{\sigma \left({X^{\prime }}_{j}\right)}-p \end{array}$$

(2) $$\begin{array}{l} N\left(d_{i},w_{j}\right)=P\left(d_{i}\right)P\left(w_{j}|d_{i}\right);P\left(w_{j}|d_{i}\right)=\\ \overset{K}{\underset{k=1}{\sum }}P\left(w_{j}|z_{k}\right)P\left(z_{k}|d_{i}\right) \end{array}$$

The structure similarity and content similarity of English learning participants are linearly fused to obtain the node pair similarity discriminant matrix, and the threshold segmentation method is used for cooperative prediction. Next, this article introduces the core module of the cooperative prediction model of English learning participants based on the fusion of representation learning and content features


(3) $$\begin{array}{l} Sd_{gain}\left(Y\right)=\frac{\sigma \left(Y\right)-avg\left(\sigma \left(Y^{\prime }\right),\sigma \left(Y^{\prime }\right)\right)}{\sigma \left(Y\right)} \end{array}$$

(4) $$\begin{array}{l} K\left(Y_{1},Y_{2},\ldots ,Y_{m}\right)=-\overset{m}{\underset{j=1}{\sum }}M\left(K_{j}\right)\log_{2}\left(P\left(K_{j}\right)\right) \end{array}$$

(5) $$\begin{array}{l} F\left(X_{f}\right)=\overset{N}{\underset{x=1}{\sum }}\frac{N_{1x}+N_{2x}+\ldots +N_{mx}}{T} \end{array}$$

In addition:


(6) $$\begin{array}{l} I\left(M_{1x},\ldots ,M_{mx}\right)=-\varphi \overset{M}{\underset{j=1}{\sum }}K_{jx}\log_{2}\left(K_{jx}\right) \end{array}$$

The topic vector distribution of English learning participants is obtained through the topic model of English learning participants


(7) $$\begin{array}{l} Q=\left\{\left(a_{1},b_{1}\right),\left(a_{2},b_{2}\right),...,\left(a_{n},b_{n}\right)\right\} \end{array}$$

(8) $$\begin{array}{l} b=\arg\max\underset{a_{n}\in W_{K}\left(a\right)}{\sum }|\left(b_{n}=c_{n}\right) \end{array}$$

Web-based English learning participants' similarity calculation, model fusion calculation and parameter selection method calculation are shown in the following formula:


(9) $$\begin{array}{l} \text{G}(\text{v},\text{e})=\left[\;{\;\zeta \;}_{1}{\text{c}}_{1}(\text{t})+{\;\zeta \;}_{2}{\text{c}}_{2}(\text{k})+{\;\zeta \;}_{3}{\text{c}}_{3}(\text{k})+{\;\zeta \;}_{4}{\text{c}}_{4}(\text{k})+{\;\zeta \;}_{5}{\text{c}}_{5}(\text{k})+{\;\zeta \;}_{6}{\text{w}}_{\text{ik}}\;\right] \end{array}$$

(10) $$\begin{array}{l} c_{1}\left(t\right)\geq 0,c_{2}\left(k\right)\geq 0,c_{3}\left(k\right)\geq 0,c_{4}\left(k\right)\geq 0,c_{5}\left(k\right)\geq 0 \end{array}$$

Given a network G (V, e), V is the vertex set, e is the edge set, | V | = n, | e | = m, let the weight value of Min be:


(11) $$\begin{array}{l} \min w_{k}\left(t\right)=\left[\omega_{1}\left(\frac{d_{k}}{V}\right)+\omega_{2}\left(\frac{d_{k}}{V}\right)+\omega_{3}\left(\frac{T_{k}}{ND_{K}}\right)+\omega_{1}\left(P_{K}T_{K}\right)\right] \end{array}$$

(12) $$\begin{array}{l} \text{W}=\vartheta^{*}=\frac{2k}{k+1}+\frac{2c_{1}+c_{2}+3et-2et\zeta }{3} \end{array}$$

The adjacency matrix of a graph is w. The degree of node I is Di, and the degree matrix of the graph is d. The classical network representation learning method is selected as the baseline method for embedded representation of English learning participants.


(13) $$\begin{array}{l} \left(In-\alpha W\right)y=\left(In-\alpha W\right)X\beta +\varepsilon \end{array}$$

(14) $$\begin{array}{l} {\text{D}}_{\kappa }=\frac{2k}{k+1}+\left[\frac{1}{2}+\frac{1}{2k}\right]{\left[\frac{c_{2}-c_{1}}{3}\right]}^{2}+\frac{2\left(c_{2}-c_{1}\right)}{3} \end{array}$$

(15) $$\begin{array}{l} \psi =\overset{\theta }{\underset{x=1}{\sum }}Vx=\overset{\vartheta }{\underset{x=1}{\sum }}\left(\frac{Wx}{\overset{n}{\underset{1}{\sum }}W_{ℑ}}Sx\right) \end{array}$$

The Stochastic Gradient descent (SGD) method is used to update the numerical value convergence


(16) $$\begin{array}{l} \Delta Q_{L}+\Delta Q_{S}+\Delta Q_{R}=\Delta Q \end{array}$$

(17) $$\begin{array}{l} w_{ik}=\overset{n}{\underset{a}{\sum }}\tau_{1}X_{ik}+\overset{n}{\underset{b}{\sum }}\tau_{2}U\left(Y_{ik}\right)+B_{ik} \end{array}$$

(18) $$\begin{array}{l} {{\text{w}}_{\text{G}}}^{\text{SGD}}=\max\left\{0,W_{G}\cdot \varepsilon \left({f_{G}}^{A_{i}},{f_{G}}^{A_{j}}\right)\right\} \end{array}$$

In deep walk model, a random walk is used to generate a vertex sequence. Each node sequence is similar to a sentence in the language, and each node pair is similar to a word. Skip gram method is used for learning and training, and the vector representation of the node is obtained.


(19) $$\begin{array}{l} I=f\left(W^{e}D_{1}+\delta^{e}\right) \end{array}$$

(20) $$\begin{array}{l} D_{a}=g\left(W^{d}I+\delta^{d}\right) \end{array}$$

The representation vectors of nodes with strong proximity are closer, and the objective of first-order optimization is


(21) $$\begin{array}{l} {f_{R}}^{A_{i}}={w_{G}}^{A_{i}A_{j}}\cdot V \end{array}$$

(22) $$\begin{array}{l} P\left(d_{i},w_{j}\right)=P\left(d_{i}\right)P\left(w_{j}|d_{i}\right);P\left(w_{j}|d_{i}\right)=\\ \overset{K}{\underset{k=1}{\sum }}P\left(w_{j}|z_{k}\right)P\left(z_{k}|d_{i}\right) \end{array}$$

(23) $$\begin{array}{l} \lambda \left(Wi,Wj\right)=\\ \left[\log\left(\frac{\left|x_{A_{i}}-a_{A_{j}}\right|}{w_{A_{j}}}\right),\log\left(\frac{\left|y_{A_{i}}-y_{A_{j}}\right|}{h_{A_{j}}}\right),\log\left(\frac{w_{A_{i}}}{w_{A_{j}}}\right),\log\left(\frac{h_{A_{i}}}{h_{A_{j}}}\right)\right] \end{array}$$

The objective of second-order optimization is as follows


(24) $$\begin{array}{l} MES\left(y,y^{\prime }\right)=\frac{\sum_{i=1}^{m}\left(y_{i}-y_{i}^{\prime }\right)}{m} \end{array}$$

Because the algorithm is simple, the result of line node prediction is not very stable and the dependence on the initial value is also serious.

## Construction of the Mode of Cooperation and Competition in English learning

### Content

This article constructs a cooperative and competitive model of English learning based on multimodal information fusion. The main performance is that students form teams in groups, and then there is a competitive relationship between groups. The competition of students in learning is the common interest of the whole group, so that each student will be more competitive for the overall interest. This article refers to the subject association model in the literature and makes the following adjustments: English grammar, vocabulary, and language sense abilities learn together in team communication and improve students' abilities in many aspects. Finally, a questionnaire was designed to investigate the participants.

### Methods and Steps

The students are divided into groups. The groups usually have four to six people. Keep the group level close. Each group member maintains a logical difference in many aspects, including personality, language, foundation, and hobbies. In particular, avoid the situation where the group members are all cross-graded and introverted students, so that the students in the group have their own characteristics, learn from each other's strengths, and help each other. At the same time, it solves the diversity and fairness of collaborative learning. This work can be done by the monitor and teacher together to promote mutual cooperation and interdependence between the team members. Then the teacher can choose the appropriate group leader for each group. Leaders need to have advantages in academic performance, interpersonal relationships, learning attitudes, organizational skills, etc. Team leaders need to have certain skills to organize English learning activities under the guidance of teachers. For example: Motivate team members to actively participate, supervise everyone to express in English, discuss topics, understand the progress of activities, etc. Create a good learning environment, stimulate students' learning motivation, and work hard in the process of collaborative learning. Team members work together for the same learning goal, communicate with each other, supervise each other, encourage each other, strengthen mutual emotions between members, and cultivate students' autonomy and learning abilities.

The traditional prediction methods based on network representation learning only consider the structural characteristics of the cooperative network of English learning participants, and do not consider the impact of the common research content between two English learning participants on scientific research cooperation. How to measure the similarity of the content between two English learning participants is also worth considering. The traditional content similarity calculation methods use one hot model, Term Frequency-Inverse Document Frequency (TF-IDF) representation, etc. When the number of words is large, it will bring the problem of dimension disaster. Based on the deductive and evaluative model of English competitive learning described in this article, this article proposes that we should pay attention to the influence of five factors on classroom communication: professional knowledge, language knowledge, strategic ability, language environment, and personal characteristics. Based on the above model, this article needs to start from these five aspects to redesign the strategies and methods used in teaching. This article also gives some specific suggestions. In recent years, the curriculum practice shows that this new teaching mode has achieved good results in activating the classroom atmosphere and promoting active learning, and has significantly improved the teaching quality and curriculum satisfaction. The cooperative learning model introduces a competitive mechanism. This type of group collaborative learning method can generate cohesion and centrifugal force between groups. Working together for the honor of the team has strengthened the spirit of unity and cooperation, and the resulting group consciousness can promote the development of usual collaborative learning activities. At the same time, this article believes that this concept and model can also be used for reference by other non-English majors. At this stage, the teaching modes adopted in this article are: students preview textbooks, fill in the experimental report, watch the teaching video in class, teachers explain the experimental steps after class experimental data processing, and complete the experimental report. The teacher scored according to the experimental operation, preview, experimental report, and other aspects. At the same time, some experimental projects were also opened. Students learned by watching Massive Open Online Courses (MOOC) video and completed the experimental report. Some students like to study independently and think that collaborative learning takes a lot of time, while others feel that they have to take courses with weak foundations. All these require authors and colleagues to continuously explore the educational process to help students to improve their ability to learn English. However, it should also be noted that the shortcomings of the learning cooperative competition model are that it may cause some unnecessary conflicts between students. The form of cooperation may cause negative emotions in individual students and affect the efficiency of English learning.

### Questionnaire Design

The sampling survey method adopts the method of random learning, and the questionnaire is distributed randomly to the survey subjects to ensure the randomness of the results. Two different scales were used in this test, one for pretest and one for the posttest, and one for the middle test. Use different perspectives to design the questionnaires for teachers and students, and consider the differences between teachers and students to ensure the wide applicability of the questionnaire. According to the content of the experiment, 15 single choice questions were designed for the pretest and posttest questions, with 1 point for each question, a total of 15 points. The midterm test is composed of 15 single choice questions and 12 subjective questions designed by the experimental content of the required stage. Because our students only need to complete four experiments at this stage, they only need to choose four of them to answer 1 point for each question, and the full score is 4 points. In this way, the measured results of students are more accurate to prevent students from being unfamiliar with other experiments, resulting in the final test data are not accurate enough. At the same time, this article also adds some subjective surveys in the second half of the questionnaire combined with cooperative learning and autonomous learning, and attaches the analytic hierarchy process scale. In particular, the main purpose of setting the midterm test questions in this project is to use the subjective test questions to screen out the students who have independently joined the appropriate cooperative learning or autonomous learning mode in the experimental process, to provide a certain range in the later data processing. The purposes of this test are: first, the type of the test is to provide a new idea for the future college English experimental test; second, to investigate the cognitive level of different levels of students in cooperative learning mode or autonomous learning mode under the framework of the new test.

## Construction of the Mode of Cooperation and Competition in English Learning

Students' learning motivation is shown in Table 1. According to Table 1, both external motivation and internal motivation are below 3 points, and the average value of internal motivation is lower than the average value of external motivation 0.12. This shows that among the respondents, internal reading motivation still dominates. The average score for unmotivated is above 3 and is higher than the average score of 1.1 for internal motivation, indicating that students still have a certain learning motivation when learning English, and they know why they want to learn English.

**Table 1:** Students' learning motivation.

**Motivation**	**Internal**	**External**	**No motivation**
**type**	**motivation**	**motivation**	**to learn**
Average value	2.76	2.88	3.86

The types of learning motivation and academic performance are shown in Table 2. The student's academic performance is closely related to the internal and external learning motivation. The internal motivation (2.52) of the students with good academic performance is significantly higher than the external motivation (3.02). There is no obvious difference between the internal motivation (2.78) and the external motivation (2.81) of the students of middle learning. Students with poor academic performance have significantly lower internal motivation (3.06) and external motivation (2.82). In general, the higher the students' internal motivation, the better their performance in learning, and *vice versa*. Internal motivation can provide students with long-term enthusiasm and motivation for learning. The English class itself is a kind of enjoyment for them, and English learning makes them feel happy.

**Table 2:** The types of learning motivation and academic performance.

**Academic performance**	**External motivation**	**Internal motivation**
Good	3.02	2.52
Medium	2.81	2.78
Difference	2.82	3.06

**Figure 4:**
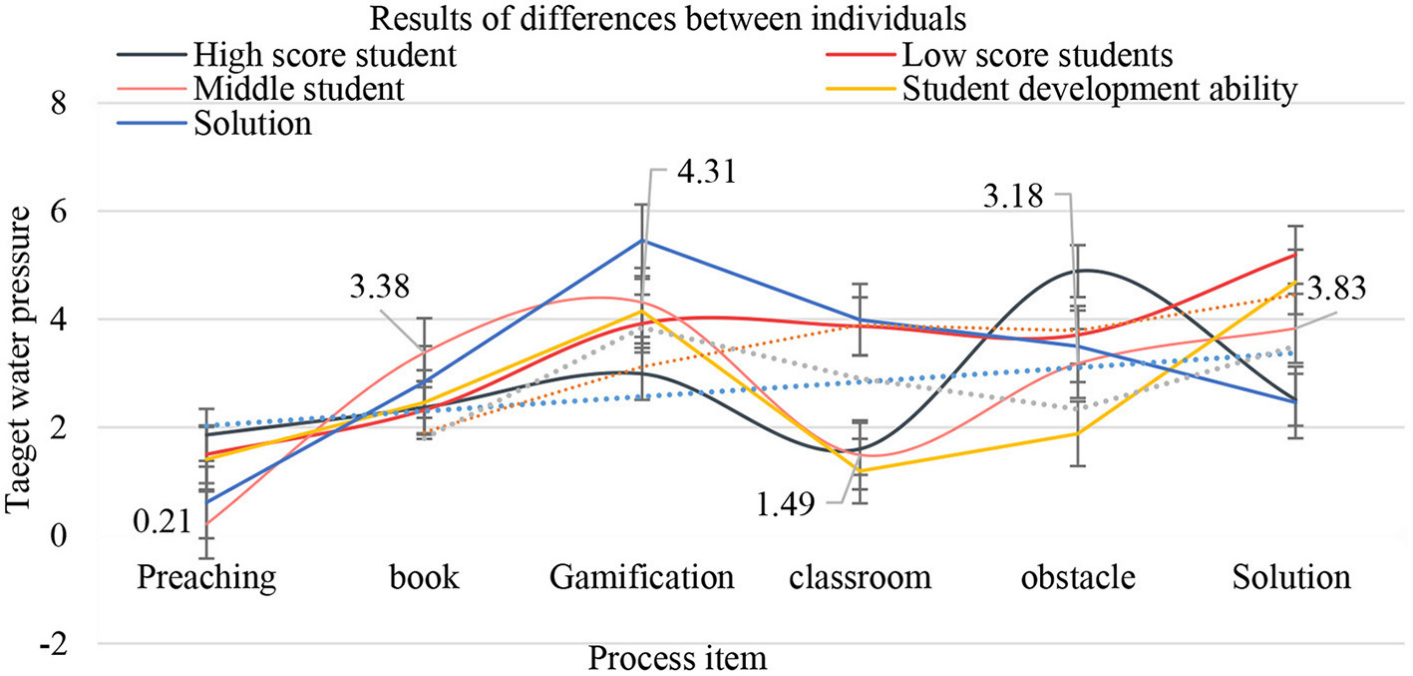
Results of differences between individuals.

As shown in Figure 4, for students with different learning abilities and grades, the same teaching method may have different effects, which is due to the differences between different individuals. With the development of science and technology, research in various fields has shown a trend of rapid differentiation and integration, and research on individual differences has also been minimized and integrated. Individuals are affected by the interaction of genetics and environment during their development, which leads to individual manifestations of various physical and psychological characteristics. Therefore, the influence of cooperative learning mode and autonomous learning mode on students' performance in different grades may be different. To explore the difference of this influence, this survey further refines the scale on the basis of the original scale of students' experimental ability, so that students only need to answer the relevant questions of their experiments at the end of the course, to improve the effectiveness of the questionnaire. At the same time, the students who have chosen the cooperative learning mode or autonomous learning mode in the experiment are selected, and they are divided into different levels according to the test scores, and then, whether cooperative learning or autonomous learning improves their scores significantly is studied.

As shown in Table 3, small-scale team is the best way to learn tacit knowledge and the most creative organizational unit. English team learning is easier to improve the overall team performance by stimulating the individual creativity of members. Moreover, in large-scale teams, the mediating path of team interaction and team cohesion has the least effect. The reason is that when students are in the English learning team, they have the least effect. When the number of members is large, the difficulty of interaction and cooperation among members increases, and the demand for team internal personnel management increases. Therefore, the team interaction process is easy to be blocked, and it is difficult to be transformed into team performance. In terms of the heterogeneity of team members' school composition, English team learning has a greater impact on team innovation performance.

**Table 3:** Small-scale teams are tacit knowledge learning.

**Item**	**High-score student**	**Low-score students**	**Middle student**	**Student development ability**	**Solution**
Preaching	1.86	1.5	0.21	1.41	0.61
Book	2.37	2.32	3.38	2.46	2.84
Gamification	2.99	3.92	4.31	4.15	5.46
Classroom	1.6	3.87	1.49	1.19	3.99
Obstacle	4.89	3.71	3.18	1.88	3.5
Solution	2.51	5.19	3.83	4.69	2.46

**Figure 5:**
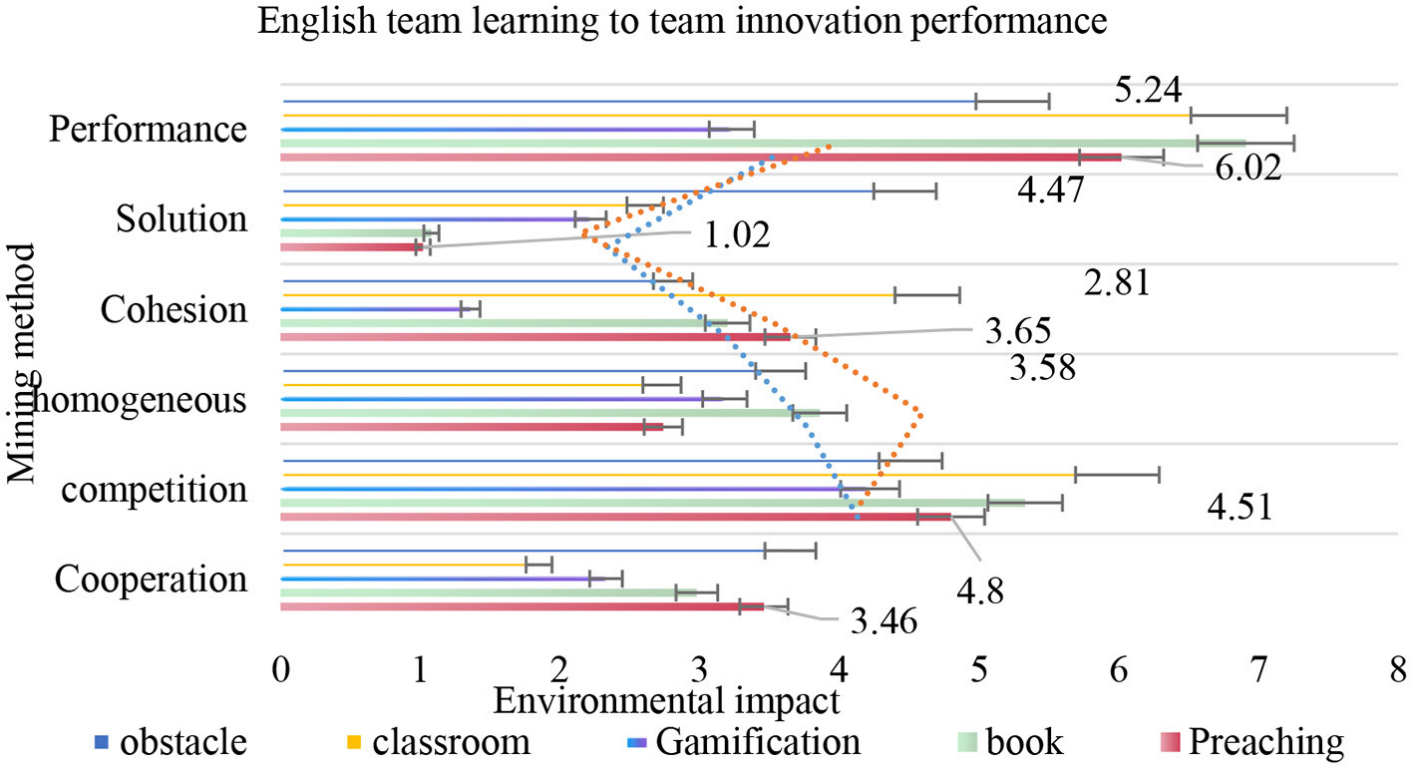
English team learning to team innovation performance.

As shown in Figure 5, whether the team members come from the same type of school or not, the direct effect of English team learning on team innovation performance is greater than the indirect effect. Comparing the contribution of different paths, it is found that the contribution rate of direct path and the mediating path through team interaction and cohesion in the heterogeneous team formed by schools is slightly less than that in the homogeneous team formed by schools; the contribution rate of mediating path through member creativity in the heterogeneous team formed by schools is greater than that in the homogeneous team formed by schools.

The results of the questionnaire are shown in Table 4. First, when the members come from different levels of colleges and universities, the interaction process and the formation of cohesion among team members are affected by background differences and lack of tacit understanding. Therefore, English team learning is more likely to affect team performance by stimulating the individual creativity of the members. Second, when the members come from the same level of colleges and universities, due to the lack of university training, it is easier to translate English team learning platform support into team performance or to influence team performance by improving team members' interaction and cohesion.

**Table 4:** Affect the interaction process and cohesion between team members.

**Item**	**Preaching**	**Book**	**Gamification**	**Classroom**	**Obstacle**
Cooperation	3.46	2.98	2.33	1.85	3.65
Competition	4.8	5.33	4.22	5.99	4.51
Homogeneous	2.74	3.86	3.18	2.73	3.58
Cohesion	3.65	3.2	1.36	4.63	2.81
Solution	1.02	1.08	2.22	2.61	4.47
Performance	6.02	6.91	3.23	6.86	5.24

**Figure 6:**
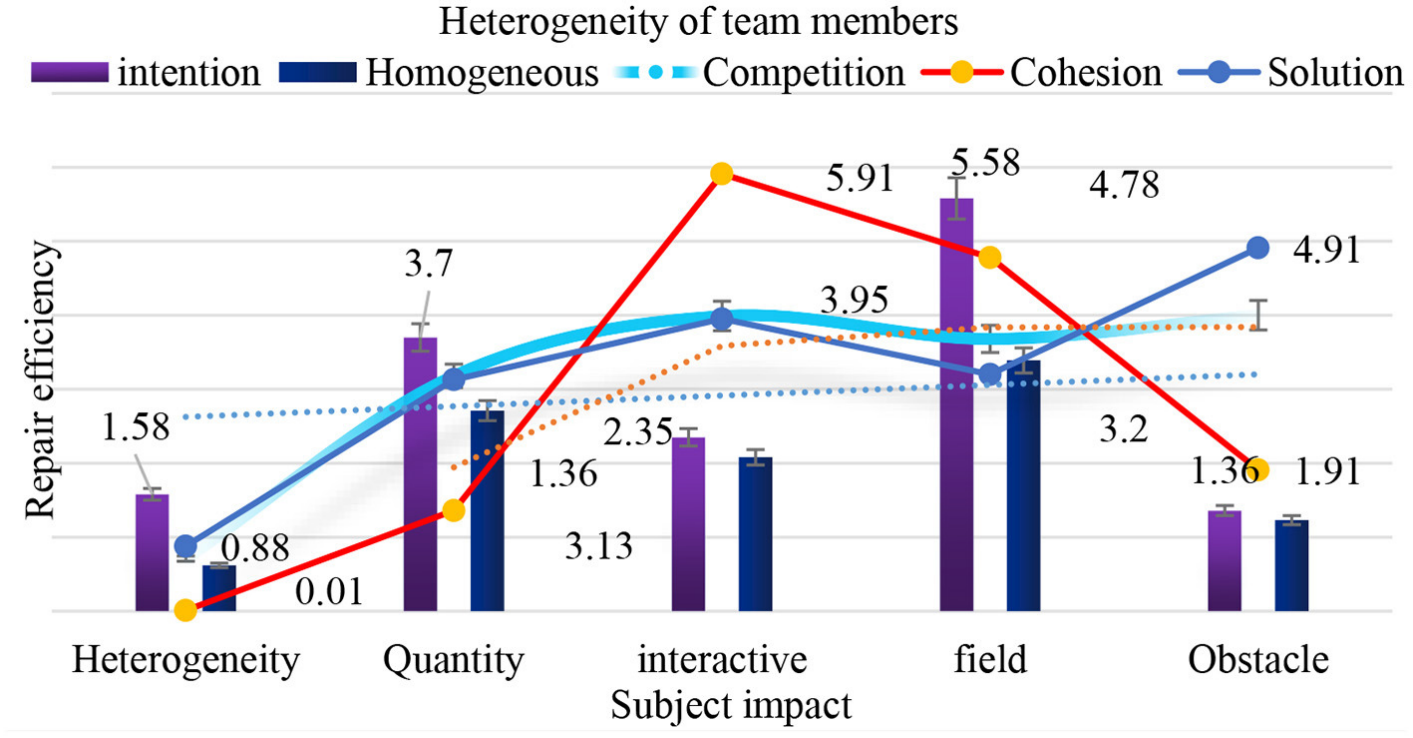
Heterogeneity of team members.

As shown in Figure 6, in terms of the heterogeneity of team members' subject composition, when the number of subjects in the team is large, the overall impact of English team learning on team innovation performance is greater, and multidisciplinary teams can promote the performance transformation of English learning space. By comparing the team path coefficients of different disciplines, it is found that the contribution rate of promoting performance transformation by improving member creativity is greater in teams with fewer disciplines than in teams with more disciplines; in teams with more disciplines, it is easier to improve team cohesion by promoting team interaction process and to promote team innovation performance transformation. The average value of the teaching method survey is shown in Table 5.

**Table 5:** The average value of the teaching method survey.

**Project**	**1**	**2**	**3**	**4**
Result	3.77	3.01	2.09	2.08

**Figure 7:**
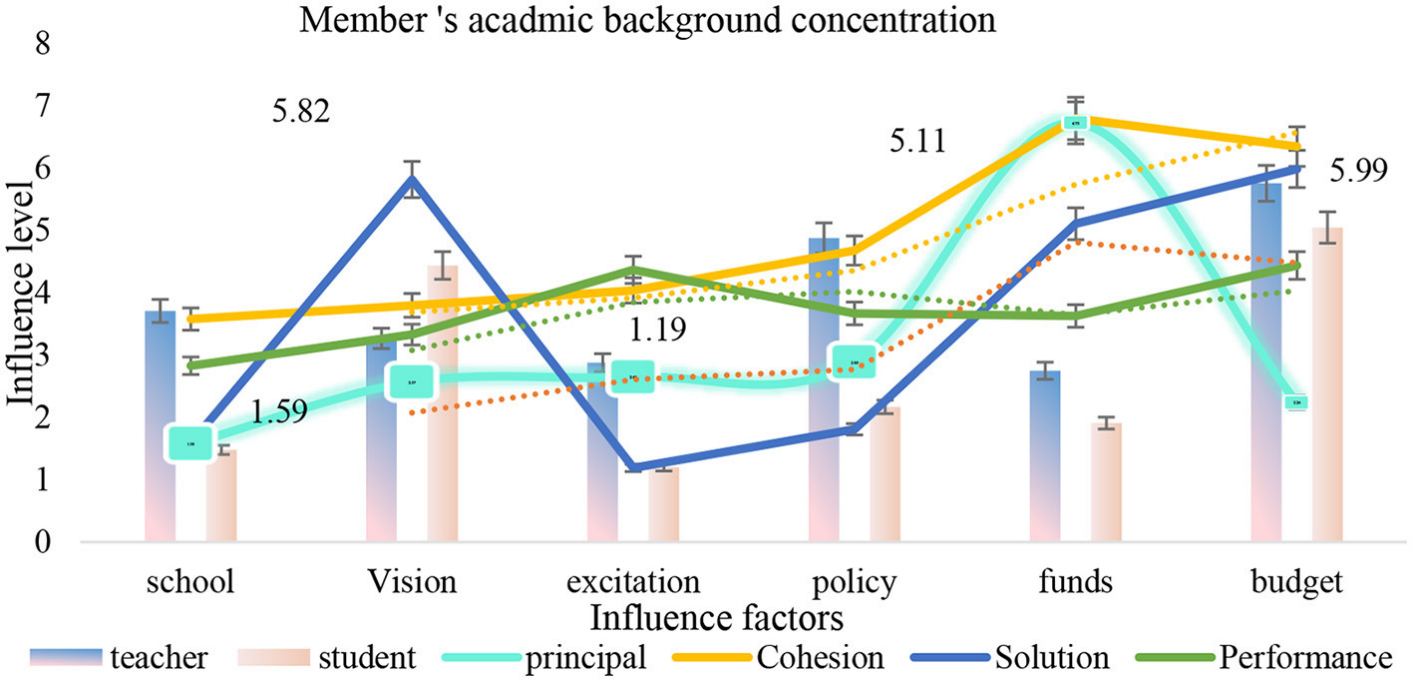
Member's academic background concentration.

As shown in Figure 7, when the number of subjects in the English learning team is small, it is easy to produce adverse effects due to the lack of voice in different professional fields; however, due to the relatively concentrated disciplinary background of the members and the team members' in-depth discussion of professional knowledge, the team mainly relies on the individual creativity of the members to promote the team's innovation performance; second, the English learning team is built-in the greater the differences in the professional fields of the members, the more they can examine and solve problems from a diversified perspective, the richer the content of ideological exchange and collision among the members, in the end, a benign interaction will be formed in the creative process, so that the process of transforming the support of the English team learning platform into team innovation performance is smoother. As shown in Table 6, English team learning, as the main carrier of innovation and entrepreneurship activities, has an important impact on the innovation performance of students' English learning teams.

**Table 6:** English team learning as an innovation and entrepreneurship activity.

**Item**	**Intention**	**Competition**	**Homogeneous**	**Cohesion**	**Solution**	**Performance**
Heterogeneity	1.58	0.71	0.62	0.01	0.88	1.71
Quantity	3.7	3.18	2.71	1.36	3.13	1.63
Interactive	2.35	3.99	2.08	5.91	3.95	3.03
Field	5.58	3.68	3.39	4.78	3.2	5.51
Obstacle	1.36	4	1.23	1.91	4.91	3.88

**Figure 8:**
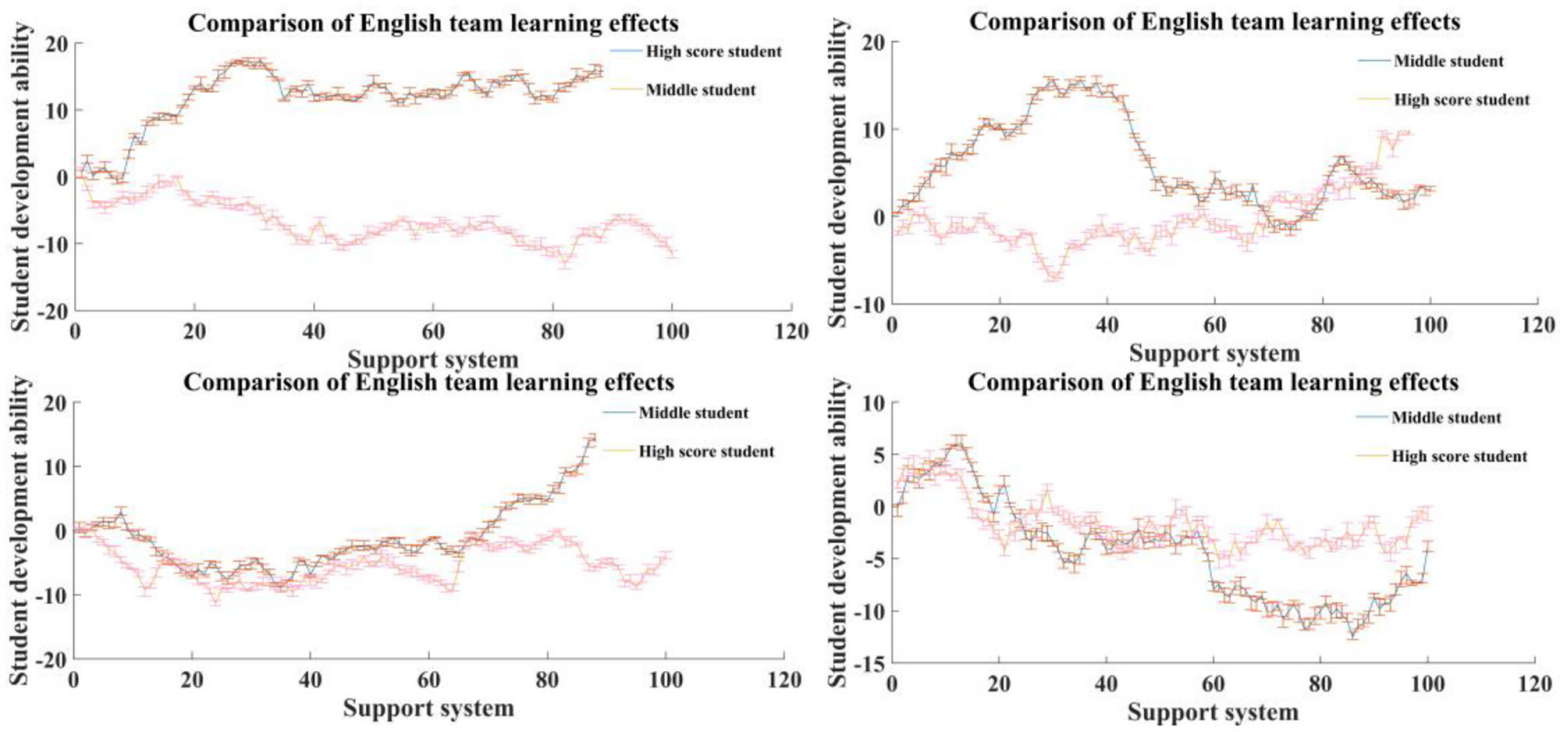
Comparison of English team learning effects.

The measurement and heterogeneity test results of English team learning and team innovation performance are shown in Figure 8. At present, the students' English learning teams feel that the software and hardware support provided by English team learning is insufficient. In the small-scale team and the team with high homogeneity of members' school sources, the common goal and vision is to create a positive creative atmosphere; while in the large-scale team and the team with high heterogeneity of members' school sources, a reasonable division of tasks can make up for the lack of common vision and create a good English team learning atmosphere. In the team with larger scale and higher homogeneity of member schools, the contribution of team innovation achievement is lower; in the team with smaller scale and higher heterogeneity of member schools, the contribution of member innovation intention is lower. The team members' subject heterogeneity has no significant effect on English team learning platform and team innovation performance.

**Figure 9:**
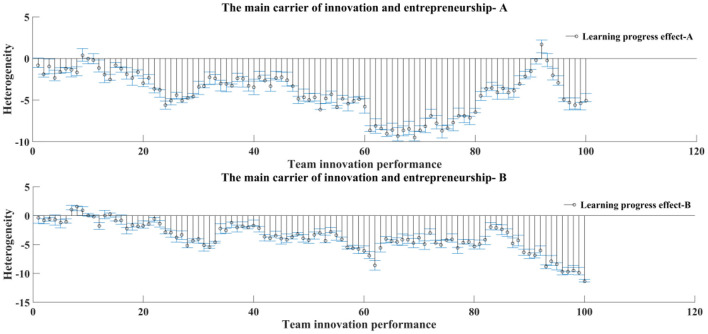
The main carrier of innovation and entrepreneurship.

Based on the heterogeneity test results of students' English learning team characteristics, as shown in Figure 9, large-scale teams are more likely to directly transform the support provided by the English team learning platform into team performance; in small-scale teams, English team learning is more likely to improve the team's overall performance by stimulating individual creativity; while in large-scale teams, the team interaction process is difficult to transform into team performance. When the source of member universities is homogeneous, it is easier to promote team performance through the positive interaction among team members, while when the source of member universities is heterogeneous, it is mainly through individual creativity to achieve team performance. When the members come from the same discipline, they mainly rely on the individual creativity of the members to promote the team innovation performance; when the team members have different professional fields, it is easy to form the positive interaction in the process of creation, and then turn into the team innovation performance.

**Figure 10:**
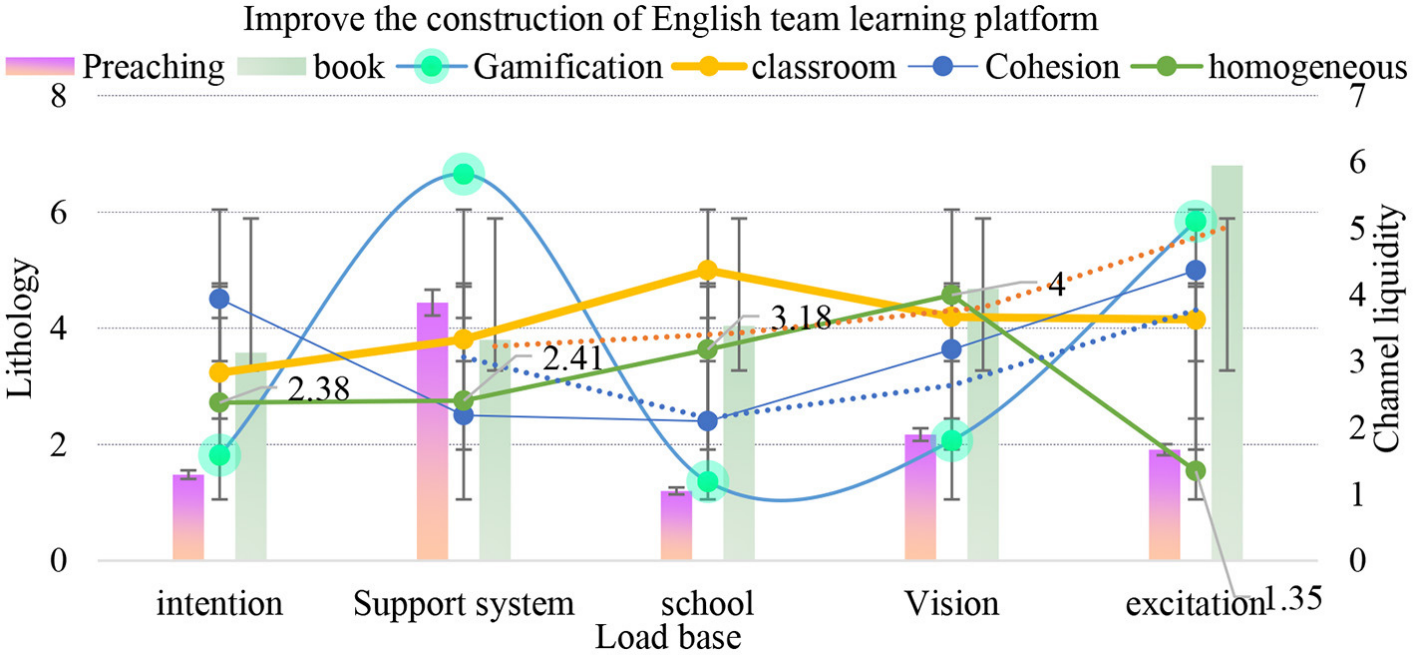
Improve the construction of English team learning platform.

As shown in Figure 10, after changing the English team learning mode and optimizing the support system of English team learning for students' English learning team, the combination mode of English learning cooperation and competition proposed in this article can improve the learning effect by 55–60%. With the vigorous promotion of national policies, English team learning, as an important carrier of innovation and entrepreneurship, has entered a stage of rapid development, but the speed of optimizing the internal quality of English team learning cannot catch up with the growth rate of the total scale. In addition to providing hardware infrastructure to ensure the normal development of daily activities of English learning teams, team learning also needs to explore the establishment of a systematic internal management systems.

As shown in Table 7, we should strengthen the influence of innovation atmosphere and English learning culture, and create an innovation atmosphere of “free innovation” by carrying out a series of activities such as lectures, entrepreneurship guidance, and exchange meetings in English team learning. At the same time, English team learning should cooperate closely with colleges and universities and improve team innovation performance through scientific selection and scientific team formation.

**Table 7:** Innovative atmosphere and the influence of English learning culture.

**Item**	**Support system**	**School**	**Vision**	**Excitation**	**Policy**	**Funds**
Heterogeneity	2.71	1.36	3.13	1.63	1.55	1.8
Quantity	2.08	5.91	3.95	3.03	4.93	2.79
interactive	3.39	4.78	3.2	5.51	2.71	2.75
field	1.23	1.91	4.91	3.88	1.85	1.46
Intention	4.13	4.28	6.82	2.07	6.56	2.9

As shown in Table 8, English team learning should be combined with the specific talent needs of team innovation and entrepreneurship projects, mining and gathering students with different advantages and talents, scientifically forming an English learning team, and scientifically determining the team size and member structure characteristics. For example, for high-quality small-scale innovation and entrepreneurship projects, we should fully explore the unique advantages of individual creativity of members, and at the same time, we should ensure the diversified member structure of the team to prevent adverse effects. After the team has entered a period of stable development, we should take advantage of the resource advantages of English learning space platform to employ well-known innovative and entrepreneurial talents, venture capitalists, and entrepreneurs to form an English team learning tutor library, to improve the team performance by improving the individual creativity of members.

**Table 8:** English team learning, team innovation, and entrepreneurship projects.

**Item**	**Heterogeneity**	**Quantity**	**Interactive**	**Field**	**Intention**
Vision	1.36	3.13	1.63	1.55	1.8
Excitation	5.91	3.95	3.03	4.93	2.79
Dimension	4.78	3.2	5.51	2.71	2.75
Policy	1.91	4.91	3.88	1.85	1.46
Funds	4.28	6.82	2.07	6.56	2.9

**Figure 11:**
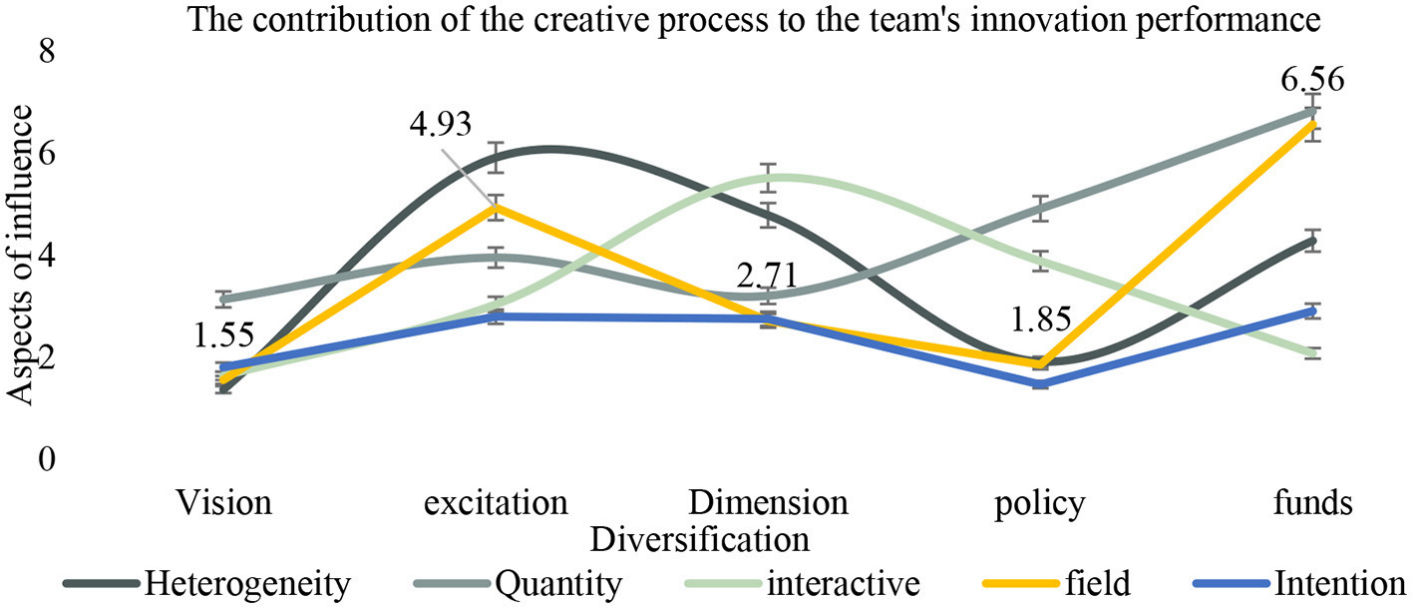
The contribution of the creative process to the team's innovation performance.

As shown in Figure 11, we should give full play to the “aggregation” role of English team learning and enhance the contribution of the creative process to team innovation performance. First of all, English team learning, as a public space for communication and resource sharing in students' English learning, should create opportunities and build a platform for positive interaction and communication and cooperation among team members by means of various theme activities such as exchange salon and entrepreneurship competition, to enhance team cohesion and help the output of innovation and entrepreneurship achievements.

As shown in Table 9, with the rapid increase in the number of English learning teams and members, it is increasingly difficult to manage and coordinate within the team. Based on the characteristics of the team, it is necessary to further explore the scientific mode of information and resource sharing among members, the institutional mechanism of team cooperation and win-win results, and the effective path of innovation and entrepreneurship achievement transformation. Finally, according to the needs of English learning teams in different stages of innovation and entrepreneurship, combined with the field of entrepreneurial tutors, through scientific guidance and planning, we can improve the ability of students' English learning teams to reasonably allocate the tasks and the enthusiasm of team members, so as to enhance the team's “joint force.”

**Table 9:** The number of teams and members in English team learning.

**Item**	**Heterogeneity**	**Quantity**	**interactive**	**field**	**Obstacle**	**Partial**	**Disorders**
Intention	2.69	5.53	3.27	2.57	4.44	3.8	5.82
Competition	4.01	5.29	2.88	2.65	1.2	4.04	1.19
Homogeneous	3.74	1.16	4.88	2.89	2.17	4.68	1.81
Cohesion	6.57	6.15	2.75	6.73	1.91	6.8	5.11
Solution	4.66	3	5.76	2.24	5.05	6.35	5.99
Performance	1.46	4.49	4.3	5.68	4.36	3.57	6.03

**Figure 12:**
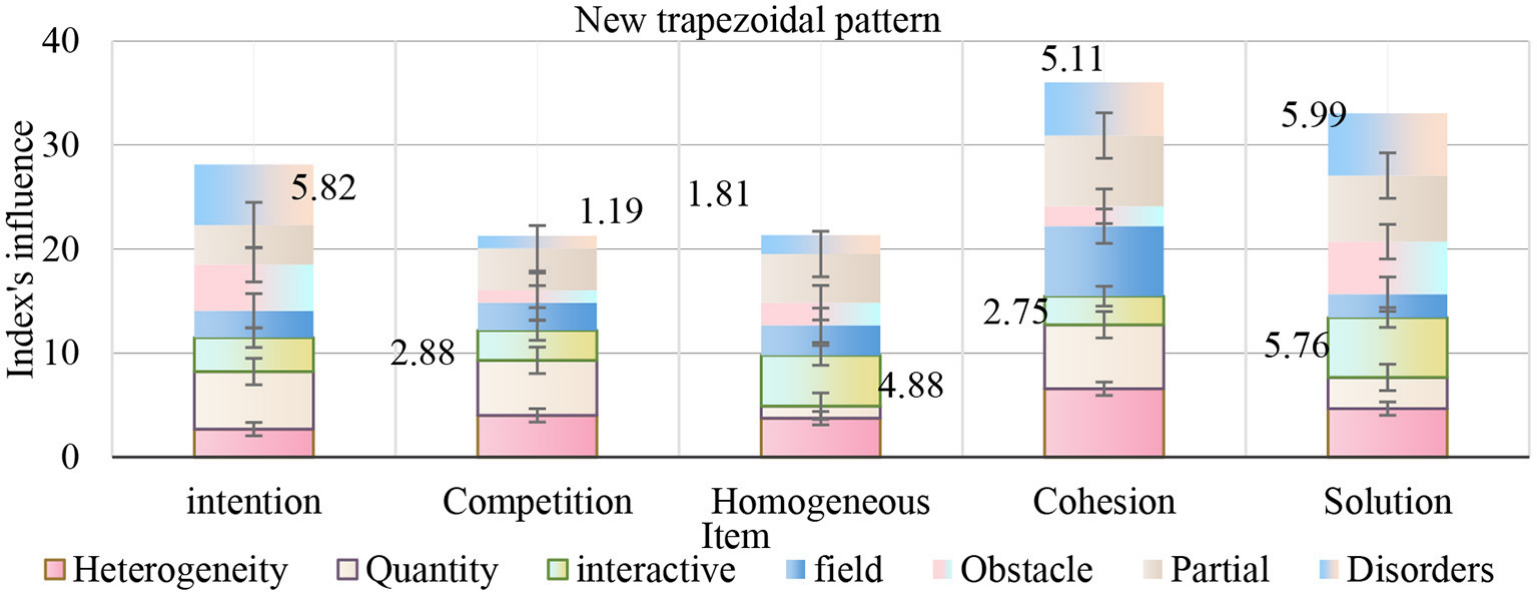
New trapezoidal pattern.

As shown in Figure 12, in view of some common problems in the current English teaching of English subjects, combined with the practical experience in the English teaching of relevant English courses in recent years and some reflections on applied linguistics, this article expounds some new opinions on the classroom communication and feedback link in the English teaching. In all English teaching, the two dimensions of professional knowledge and English ability training are not orthogonal and mutually exclusive, but should be mutually supportive and dependent. To form an effective teaching mode of “student-centered and teacher-led,” active and rich communication and feedback in the classroom are the keys, which also help to form a gradual cycle of teaching and learning.

At the beginning of the semester, four monitors of the experimental class and the comparison class were called to distribute the questionnaire forms and explain related matters to obtain relevant data. The survey results are shown in Table 10. It can be seen from the table that the average score of each class varies greatly, and the average scores of student motivation and teacher self-evaluation are relatively low.

**Table 10:** Survey results.

**Class**	**Evenly divided into classes**	**Average score of student motivation**	**Teachers' self-evaluation**
1	75.4	79.5	90
2	75.1	85.0	90
3	52.7	65.5	70
4	47.6	60.9	75

**Figure 13:**
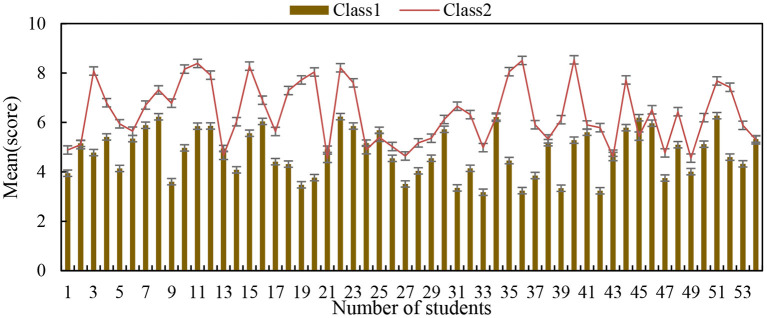
Pretest results comparison between the experimental class and the control class.

Figure 13 shows the pretest results comparison between the experimental class and the control class. The average score of the experimental class is 0.16 lower than that of the control class (the two classes are 8.59 and 8.75, respectively), and the *T* value is −0.164, which is for below 5% of the theoretical critical value of 1.69. Therefore, we can conclude that there is no significant difference between the two classes in the pretest results.

**Figure 14:**
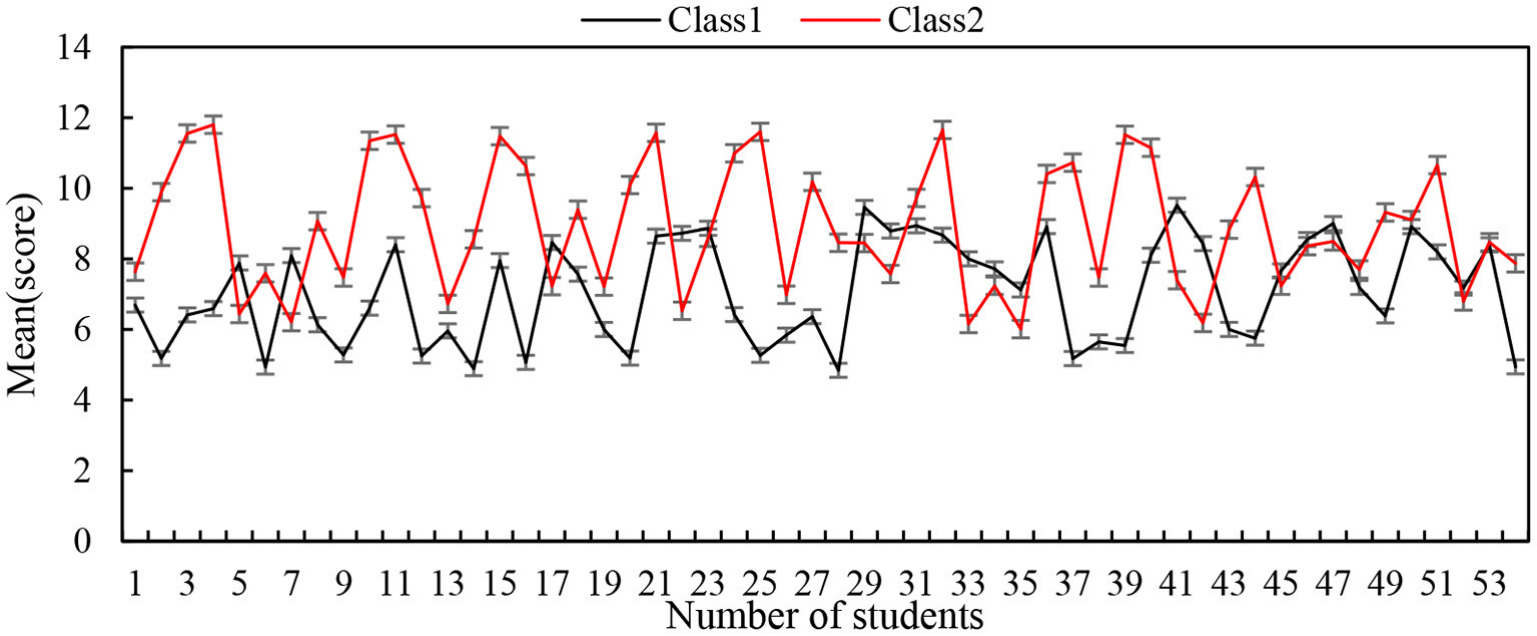
The test results of the two classes after the experiment.

The test results of the two classes, after the experiment, are shown in Figure 14. The average number of the experimental class is 1.88 higher than that of the control class (the two classes are 14.25 and 12.37, respectively), and the *T* value is 2.1, which is significantly higher than 5% of the theoretical critical value of 1.69. From this, we can see that the cooperative learning mode in the experimental class played an exact role and achieved remarkable results, thus verifying that the cooperative learning model is significantly better than the traditional teaching method.

## Conclusions

At present, students' English learning teams feel that the software and hardware support provided by English team learning is insufficient. In the small-scale team and the team with high homogeneity of members' school sources, the common goal and vision create a positive creative atmosphere; while in the large-scale team and the team with high heterogeneity of members' school sources, a reasonable division of tasks can make up for the lack of common vision and create a good English team learning atmosphere. In the team with larger scale and higher homogeneity of member schools, the contribution of team innovation achievement is lower; in the team with smaller scale and higher heterogeneity of member schools, the contribution of member innovation intention is lower. Team members' subject heterogeneity has no significant effect on English team learning platform and team innovation performance.

By changing the English team learning model and optimizing the English team learning support system of the students' English learning team, the English learning model based on multimode information fusion combining cooperation and competition proposed in this article can improve the learning effect by 55–60%. With the vigorous advancement of national policies, English team learning has entered a stage of rapid development as an important carrier of innovation and entrepreneurship, but the optimization speed of the internal quality of English team learning has not kept up with the growth rate of the overall scale. In addition to providing hardware infrastructure to ensure the normal development of the daily activities of the English learning team, team learning also needs to explore the establishment of a systematic internal management system.

In the process of learning English, the training process needs to spend more time and money, which greatly increases the difficulty and cost of coordination and management, thus, further reducing the willingness and efficiency of cooperative innovation. In the aspect of trust building, people will react more negatively to opinions and behaviors that do not conform to the existing norms. When the distance between the informal institutions is large, the innovative behaviors and opinions of the two sides often deviate from each other's informal institutional norms. Therefore, it is difficult to establish a high sense of trust and identity in learning English, which seriously hinders mutual knowledge sharing and learning, and is not conducive to the development of cooperative innovation. This article tests that team achievement goal orientation can effectively predict team learning creativity, which not only promotes the development of team achievement goal theory in the field of learning creativity, but also expands the research scope of antecedents of team learning creativity. The next research will focus on the important role of team situation factors in the formation of team learning creativity, and reveal the dynamic mechanism of team learning creativity from the perspective of positive psychology.

## Data Availability Statement

The original contributions presented in the study are included in the article/supplementary material, further inquiries can be directed to the corresponding author/s.

## Ethics Statement

Ethical review and approval was not required for the study on human participants in accordance with the local legislation and institutional requirements. The patients/participants provided their written informed consent to participate in this study.

## Author Contributions

HT is responsible for conceptualization, methodology, software data curation, writing, contributed to manuscript revision, read, and approved the submitted version.

## Conflict of Interest

The author declares that the research was conducted in the absence of any commercial or financial relationships that could be construed as a potential conflict of interest.

## Publisher's Note

All claims expressed in this article are solely those of the authors and do not necessarily represent those of their affiliated organizations, or those of the publisher, the editors and the reviewers. Any product that may be evaluated in this article, or claim that may be made by its manufacturer, is not guaranteed or endorsed by the publisher.
